# Neural Responses during Social and Self-Knowledge Tasks in Bulimia Nervosa

**DOI:** 10.3389/fpsyt.2013.00103

**Published:** 2013-09-12

**Authors:** Carrie J. McAdams, Daniel C. Krawczyk

**Affiliations:** ^1^Department of Psychiatry, The University of Texas Southwestern Medical Center, Dallas, TX, USA; ^2^School of Behavioral and Brain Sciences, Center for Brain Health, The University of Texas at Dallas, Dallas, TX, USA

**Keywords:** mentalization, identity, theory of mind, eating disorders, anorexia, bulimia, neuroimaging, social behavior

## Abstract

Self-evaluation closely dependent upon body shape and weight is one of the defining criteria for bulimia nervosa (BN). We studied 53 adult women, 17 with BN, 18 with a recent history of anorexia nervosa (AN), and 18 healthy comparison women, using three different fMRI tasks that required thinking about self-knowledge and social interactions: the Social Identity task, the Physical Identity task, and the Social Attribution task. Previously, we identified regions of interest (ROI) in the same tasks using whole-brain voxel-wise comparisons of the healthy comparison women and women with a recent history of AN. Here, we report on the neural activations in those ROIs in subjects with BN. In the Social Attribution task, we examined activity in the right temporoparietal junction (RTPJ), an area frequently associated with mentalization. In the Social Identity task, we examined activity in the precuneus (PreC) and dorsal anterior cingulate (dACC). In the Physical Identity task, we examined activity in a ventral region of the dACC. Interestingly, in all tested regions, the average activation in subjects with bulimia was more than the average activation levels seen in the subjects with a history of anorexia but less than that seen in healthy subjects. In three regions, the RTPJ, the PreC, and the dACC, group responses in the subjects with bulimia were significantly different from healthy subjects but not subjects with anorexia. The neural activations of people with BN performing fMRI tasks engaging social processing are more similar to people with AN than healthy people. This suggests biological measures of social processes may be helpful in characterizing individuals with eating disorders.

## Introduction

Bulimia nervosa (BN) is an eating disorder characterized by frequent binge-eating followed by purging behaviors in concert with a self-esteem that is overly associated with body shape and weight (1). The symptoms of many eating disorder patients change during their lives (2, 3). For example, a patient may develop restricting behaviors with weight loss in high school, begin binging and purging behaviors at a low weight, continue binge-purge behaviors at a healthy weight throughout college, and then cease the purging behaviors but have occasional binge-eating problems. Such a patient would have met criteria for anorexia nervosa (AN), restricting subtype initially, then AN, binge-purge subtype, then BN, and finally binge-eating disorder. This diagnostic instability makes clinical treatment as well as research into eating disorders challenging (4, 5). A better understanding of biological and cognitive similarities and differences that contribute to eating disorders may improve clinical treatment. Currently, treatment of BN leads to sustained recovery in only about half of the patients (6, 7). Through the use of fMRI, we examined neural activations related to social processes in BN.

A specific set of neural regions is modulated in response to tasks that require thinking about people in healthy subjects (8, 9); this provides a framework to assess differences related to psychiatric illnesses. Severe impairments in social interaction are one of the diagnostic criteria for autistic spectrum disorders (1), but problems in social cognition have been reported in many psychiatric illnesses (10–14). Decreased social cognition has been reported in a variety of behavioral tasks in adults with AN (15–17). In BN, recent studies have concluded that there was little evidence of social cognition differences in psychological tasks (18, 19), although far fewer studies of social cognition have been completed in BN than in AN.

Because both BN and AN include in their diagnostic criteria an association between appearance and self-esteem (1), these experiments focused on neural pathways related to thinking about oneself. Self-esteem is a term used to describe one’s overall sense of one’s own value as a person, and is generally considered a fairly stable psychological characteristic (20). Although the diagnostic criteria in eating disorders connect self-esteem specifically to physical appearance, similarly unrealistic social expectations are reported and observed in eating disorder patients (21, 22). Self-knowledge, as used in MRI tasks, relates to the ability to evaluate oneself, and is expected to be a process that involves self-esteem as well as other criteria. For example, an individual whose self-esteem is highly related to appearance might be very good at her work, and correctly describe herself as a competent employe, but maintain an overall low self-esteem because of perceived inadequacies of appearance. Furthermore, low self-esteem has been related to prognosis in AN (23, 24) as well as onset of bulimic symptoms (25). Negative beliefs about one’s self, unrelated to physical appearance, have been observed in eating disorders (26, 27), and neural differences in the processing of these negative self-beliefs have been seen in BN (28). These data show not only that psychological similarities in self-esteem are present in AN and BN, but also that self-esteem is an important factor in assessing the prognosis and severity of eating disorders.

In healthy people, midline cortical structures, including the cingulate (Cing), dorsal anterior cingulate (dACC), and precuneus (PreC), have been specifically associated with thinking about oneself, using a variety of self-knowledge, appraisal, and viewing tasks (29). Most commonly, these areas show activation during neuroimaging tasks that ask healthy subjects to reflect upon whether specific characteristics describe oneself (30). Performance of this type of task is likely to acutely stimulate similar cognitive processes as those that generate one’s longer-term sense of self-esteem. We recently reported differences in brain activations in AN and CN based on differences in self-knowledge using two neuroimaging tasks that required self-evaluations, one using social adjectives and the other physical descriptors (31). In that study, we identified regions in the dACC, PreC, and Cing with different activations in subjects with AN compared to the healthy controls. Here, we consider the responses of subjects with BN in the same self-evaluative tasks.

In addition to self-evaluative tasks, we included a more general social processing neuroimaging task that robustly engages additional regions in the social processing network associated with considering other people (32). This task, the Social Attribution Task, strongly activates the right temporoparietal junction (RTPJ), a region that has been closely associated with theory of mind (TOM), and mentalization [for reviews, see (9, 33)], as well as the fusiform gyrus, a region closely associated with facial processing (34, 35). Furthermore, differences in fMRI activations in both adult participants with AN (36) as well as adolescent participants with AN (37) have been examined using this task. In this manuscript, we describe the neural activations of subjects with BN during the Social Attribution Task.

## Methods and Materials

### Ethics statement

This study was approved by the institutional review boards at both the University of Texas Southwestern Medical Center and The University of Texas at Dallas. Additionally, the study adhered to the guidelines as set out in the Declaration of Helsinki. Written informed consent was required from all participants, and subjects were reimbursed for time spent participating.

### Participants

A total of 53 female participants, between 18 and 42 years of age, were recruited for this study from the general public, from treatment providers, and support groups in the Dallas–Fort Worth area. Subjects volunteered to spend 2 h in clinical assessments and completing questionnaires and 1.5 h completing behavioral tasks in the MRI scanner, and were compensated for their time financially. The participant groups consisted of 18 healthy controls (CN), 18 individuals with a recent history of anorexia but were currently in the process of recovering from AN, and 17 individuals recovering from BN. All AN and BN participants had met full DSM-IV criteria for either AN or BN within the previous 2 years. The AN subjects were required to be maintaining a minimum BMI of 17.5 with no weight loss for the 3 months preceding the MRI scans. This was based primarily on a detailed eating disorder symptom and weight history obtained at the initial screening interview, only after which was it divulged that low or unstable weight was an exclusion factor for MRI scans. One of the AN and one of the CN participants did not complete the Social Attribution task and one of the BN participants did not complete the Social and Physical Identity neuroimaging task; these subjects were excluded in the analyses involving those tasks. Another BN participant was excluded from the neuroimaging analyses of the Social and Physical Identity tasks due to excessive movement. Eleven of the AN subjects had the restricting subtype and seven had the binge-purge subtype of AN. All subjects were recruited and scanned between 2009 and 2012. Because of difficulty recruiting BN subjects, data collection of AN and CN subjects finished nearly 1 year before the last four BN subjects were obtained. Therefore, the AN and CN data were analyzed and published in two earlier papers, one describing results obtained from the Social Attribution task and the other results from the two Identity tasks (31, 36).

Subjects provided written informed consent to participate in this study at an initial appointment. All subjects were then interviewed using the Structured Clinical Interview for DSM-IV disorders (SCID-RV). Participants were also screened for MRI compatibility. Some of the subjects had a history of recurrent MDD (1, CN; 7, AN; 9 BN) but none had met symptom criteria for an MDE for at least 3 months prior to the neuroimaging studies. No participants had a current or past diagnosis of any psychotic disorders or bipolar disorder based on the SCID-RV; no participants were currently taking mood-stabilizers, antipsychotics, or benzodiazepines. Participants on antidepressants whose dosage had not changed for at least 3 months prior to their MRI scans were included (1 CN; 8 AN; 7 BN).

Participants also completed the Quick Inventory of Depression, Self-Report (QIDS-SR), a self-report questionnaire consisting of 16 items to assess current symptoms of depression (38), and the Eating Attitudes Test-26 (EAT-26), a self-report questionnaire consisting of 26 items that relate to current eating behaviors (39). Subjects also completed the Self-Liking and Self-Competence Self-Esteem Questionnaire (SLCS), a 16 item self-report questionnaire that provides two measures of self-esteem (40), and the Social Problem-Solving Inventory (SPSI-R), a 26 item self-report questionnaire (41).

### Neuroimaging tasks

Three fMRI tasks were employed, the Social Attribution Task, the Social Identity task, and the Physical Identity task. Most subjects (35 of 53) preferred to complete the tasks in two scanner sessions, the first session consisting of the Social Attribution Task, and the second both the Identity Tasks. If all tasks were completed in 1 day, the Identity tasks were run before the Social Attribution task, and the total scan time was 80 min. When completed on separate days, the first day lasted about 30 min and the second session was about 50 min.

The Social Attribution Task presented short videos of moving shapes (32, 36). Briefly, subjects were asked to view the shapes in two conditions: the visuospatial or Bumper condition, preceded by the question “Bumper cars: Same weight?” and the social attribution or People condition, preceded by the question “People: All friends?”. Each animation consisted of a moving display of three white shapes (circle, triangle, and square) and a white box with one side that opened as if hinged on a black background. Although the same shapes were presented in both conditions, the movements of the shapes in the two tasks differed. During the visuospatial task, the shapes moved around the box for the duration of the animation periodically bumping into one another. During the social task, the shapes moved in ways that suggested social behavior was occurring among the shapes (e.g., playing, fighting, avoiding etc.). We recorded responses to the weight and friendship questions about the animations to determine accuracy and maintenance of concentration.

The Identity tasks consisted of the presentation of written appraisal statements projected onto a screen within the MRI scanner (31). For both the Social Identity and the Physical Identity task, three different types of appraisals were shown: self (evaluation of an attribute about one’s own identity based on one’s own opinion), Friend (evaluation of an attribute about a close female friend), and Reflected (evaluation of an attribute about one’s self from one’s friend’s perspective). Each statement was presented above a scale reading 1 “Strongly Disagree,” 2 “Slightly Disagree,” 3, “Slightly Agree,” and 4 “Strongly Agree.” Subjects were asked to read each statement and select a rating via a hand-held button. The Friend and Reflected statements were personalized to contain the name of a specific female friend of each subject. Each task was conducted separately, with all runs of the Social task preceding any runs of the Physical task. In the Social task, the statements were presented in a format ending with a socially descriptive adjective (ex. Self Statement “I believe I am nice,” Friend statement “I believe my friend is mean,” Reflected statement, “My friend believes I am responsible”). For the Physical task, the statements were presented in the format ending with a physical body part and a descriptor (ex. Self statement “I believe my arms are toned,” Friend statement “I believe my friend’s eyes are bloodshot,” Reflected statement “My friend believes my stomach is flabby”). In all cases my friend was replaced with the name of a close female friend of the subject.

### MRI acquisition and analysis

All images were acquired with a 3T Philips MRI scanner. High resolution MP-RAGE 3D T1-weighted images were acquired for anatomical localization with the following imaging parameters: repetition time (TR) = 2100 ms, echo time (TE) = 3.7 ms; slice thickness of 1 mm with no gap, a 12°flip angle, and 1 mm^3^ voxels. For both fMRI tasks, each slice was acquired with a 22.0 cm^2^ field of view, a matrix size of 64 × 64, and a voxel size of 3.4 mm × 3.4 mm × 3 mm using a one-shot gradient T2*-weighted echoplanar (EPI) image sequence sensitive to blood oxygen level-dependent (BOLD) contrast. Head motion was limited using foam head-padding.

For the Social Attribution task, images were acquired during four runs, each lasting 128 s and presenting four 17-s videos, two in each condition (People or Bumper). These sequences were acquired using a TR of 1.5 s, an TE of 25 ms, and a flip angle of 60°, and volumes were composed of 33 tilted axial slices (3 mm thick, 1 mm slice gap) designed to maximize whole-brain coverage while minimizing signal dropout in the ventral anterior brain regions. For the Identity tasks, images were acquired during eight runs (four for Social and four for Physical), each lasting 360 s, and presenting 12 statements of each condition (Self, Friend, and Reflected). These sequences were acquired using a TR of 2 s, an TE of 35 ms, and a flip angle of 0°, and volumes were composed of 36 axial slices (4 mm thick, no gap).

Prior to statistical analyses, preprocessing for all tasks consisted of spatial realignment to the first volume of acquisition, normalization to the MNI standard template, and spatial smoothing with a 6 mm 3D Gaussian kernel. fMRI task data were analyzed using Statistical Parametric Mapping software (SPM5, Wellcome Department of Imaging Neuroscience London)^1^ run in MATLAB 7.4^2^, and viewed with xjview^3^.

The fMRI data were analyzed separately for each of the three tasks. For the Social Attribution task, the data were analyzed using a general linear model to create contrast images with a block design (blocks: People and Bumper); the Identity tasks were analyzed separately using an event-related design, in which each type of event (events: Self, Friend, and Reflected) corresponded to the BOLD signal during the 4 s presentation of each statement. With both techniques, the general linear model was used to create contrast images with activation of each condition assessed using a multiple regression analysis set as boxcar functions. Each regressor was convolved with a canonical hemodynamic response function (HRF) provided in SPM5 and entered into the modified general linear model of SPM5. Parameter estimates (e.g., beta values) were extracted from this GLM analysis for the regressors. Resulting single-subject one-sample *t*-test contrast images were created for each participant for each of the three tasks. These contrast images were combined for group map analyses.

### Regions of interest

Previously we identified four regions showing group differences with whole-brain voxel-wide comparisons of the AN and CN group maps using the contrasts of conditions in the three tasks (31, 36). These were the *a priori* regions of interest (ROI) for this study focusing on BN. In the Social Attribution Task, the whole-brain voxel-wide comparisons of the AN and CN groups led to identification of a 94 voxel region in the RTPJ (MNI 52, −64, 20) that showed more modulation in the People condition than the Bumper condition in the CN subjects compared to the AN subjects. In the Social Identity Task, the whole-brain voxel-wide comparisons of the Reflected–Self contrast for the AN and CN groups led to identification of a 379 voxel region in the dACC (MNI 6, 26, 36) with the opposite modulation in the CN subjects compared to the AN subjects. In the Social Identity Task, the whole-brain voxel-wide comparisons of the Self–Friend contrast for the AN and CN groups led to identification of a 43 voxel region in the PreC (MNI −8, −48, 46) with more modulation in the Self condition than the Friend condition in the CN subjects compared to the AN subjects. In the Physical Identity Task, the whole-brain voxel-wide comparisons of the Self–Friend contrast for the AN and CN groups led to identification of a 61 voxel region in a ventral region of the dACC adjacent to the corpus callosum (cc-dACC, MNI −6, 20, 24) showing more modulation in the CN subjects than the AN subjects. In addition to the ROIs defined by group differences in these tasks, we also examined activations in medial prefrontal cortex (MPFC; vmPFC) and dorsolateral prefrontal cortex (DLPFC) based on prior reports of differences in these areas with similar tasks in eating disorder subjects. We created 5 mm spherical ROIs centered on the published coordinates for MPFC [10, 64, 18, (37)], vmPFC [−12, 44, −12, (42)], and DLPFC [−48, 6, 38 (28)]. For all ROI analyses, we extracted the percent signal change occurring within each of these regions for each subject using the MarsBar toolbox^4^ and transferred this data to (SPSS, Inc., Chicago). In SPSS, we first conducted a three-group ANOVA to identify whether differences were present across the three subject groups for each ROI, and conducted follow-up analyses of significant results using between-group *t*-tests.

## Results

### Psychological scales and demographic data

The three groups were not significantly different in age or years of education. The AN group had a significantly lower body mass index than either the CN and BN groups (Table 1). The BN and AN groups both scored higher than the CN group on measures of depression and eating behaviors but were not significantly different from each other. The AN and BN subjects also reported lower levels of both self-liking and self-competence compared to the CN subjects. On the SPSI-R, the AN and BN groups had lower overall scores on social problem solving as well as lower levels of positive problem orientation and higher levels of negative problem orientation than the CN groups. The AN subjects also showed higher levels of avoidance than the CN subjects, whereas the BN subjects had lower levels of rational-problem solving than the CN subjects. However, there were no significant differences in the AN and BN groups in comparisons for any of the SPSI-R subscales.

**Table 1:** **Sociodemographic and symptom scale values for the participants**.

	Healthy control (*n* = 18)	Anorexia nervosa (*n* = 18)	Bulimia nervosa (*n* = 17)	Between group comparisons^c^
Average age (years)	24.5 (18–39)^a^	26.1 (18–40)	28.1 (19–42)	No differences
Mean years of education	15.8 (14–20)	14.9 (12–19)	15.8 (13–18)	No differences
Current body mass index^b^	23.2 (18–35)	19.6 (18–23)	22.1 (19–27)	1 > 2, *p* = 0.003; 3 > 2, *p*=0.001
Quick inventory of depression	3.7 (0–9)	8.5 (2–17)	6.8 (1–16)	2 > 1, *p* < 0.001; 3 > 1, *p* = 0.012
Eating attitudes test	4.3 (0–15)	27.4 (1–61)	18.8 (2–51)	2 > 1, *p* < 0.001; 3 > 1, *p* = 0.001
Self liking from SLSC^d^	30.7 (19–40)	17.2 (8–29)	20.4 (8–32)	1 > 2, *p* < 0.001; 1 > 3, *p* = 0.02
Self competence from SLSC^d^	30.2 (24–40)	24.7 (18–37)	27 (17–38)	1 > 2, *p* < 0.001; 1 > 3, *p* = 0.04
Social problem solving inventory	15.3 (12–18)	12.1 (6–18)	11.6 (6–14)	1 > 2, *p* < 0.001; 1 > 3, *p* < 0.001
Positive problem orientation	13.7 (7–18)	10.4 (3–19)	10.3 (3–15)	1 > 2, *p* = 0.02; 1 > 3, *p* = 0.02
Negative problem orientation	3.9 (0–8)	10.8 (3–16)	9.2 (1–20)	2 > 1, *p* < 0.001; 3 > 1, *p* = 0.001
Rational problem solving	13.2 (6–18)	10.8 (0–16)	8.3 (3–17)	1 > 3, *p* = 0.007
Avoidance style	3 (0–14)	6.6 (0–17)	5.5 (0–13)	2 > 1, *p* = 0.028
Impulsivity/careless style	3.4 (0–10)	3.6 (0–15)	5.9 (0–16)	No differences

^a^All entries under the subject groups contain the mean (range).

^b^Mean and range for AN subjects exclude one higher weight outlier.

^c^Statistical values obtained using a three group ANOVA for each metric; *p* values provided for significant differences (<0.05).

^d^SLSC: self-Liking and self-competence scale.

### Social attribution task

The Social Attribution Task required subjects to respond to a question about each video. There were no differences in the accuracy of the subjects in response to either the Bumper visuospatial-weight question [mean percent correct, CN 59%, AN 64%, BN 69%, *F*(50) = 2.17, *p* = 0.13], or the People social-friendship question [mean percent correct, CN 77%, AN 81%, and BN 84%, *F*(50) = 1.67, *p* = 0.20]. There were also no differences in reaction times for subjects in either task [Bumper, mean reaction time in seconds, CN 1.22, AN 1.30, BN 1.35, *F*(50) = 0.28, *p* = 0.76; People, mean reaction time in seconds, CN 1.30, AN 1.35, BN 1.40, *F*(50) = 0.15, *p* = 0.86].

The People–Bumper contrast of the Social Attribution Task resulted in significant clusters of activation in the middle temporal gyri, and temporoparietal junctions (TPJ) in all three groups (Table 2). Additionally, the CN subjects had bilateral activations in inferior frontal gyri, the fusiform gyri, the medial frontal gyrus, and the PreC. The AN and BN subjects also had activations in the right inferior frontal gyrus, but not the left inferior frontal gyrus. The AN subjects also showed modulation of the medial frontal gyrus like the CN subjects, and also activated a region in the ventral anterior Cing. The BN subjects did not modulate MPFC, like the CN and AN groups, but did modulate the PreC and the fusiform gyri, like the CN subjects but differing from the AN subjects.

**Table 2:** **Clusters in the CN, AN, and BN group maps during the People–Bumper contrast of the social attribution task**.

Group	Region	Volume	Cluster *P*	Peak *T*	MNI *x*, *y*, *z*
CN	Temporal	4366	0	11.66	60, −8, −16
CN	Inferior frontal	769	0	10.9	54, 28, 8
CN	Inferior frontal	313	0	10.67	−52, 22, −4
CN	Temporoparietal	2273	0	9.07	−60, −44, 12
CN	Temporal	918	0	8.64	−54, 0, −20
CN	Cerebellum	281	0	7.47	−10, −80, −46
CN	Fusiform	764	0	7.39	44, −34, −20
CN	Precuneus	609	0	6.68	8, −46, 36
CN	Prefrontal	344	0	6.13	4, 56, 14
CN	Caudate	81	0.036	5.77	−6, 2, 4
CN	Precentral	151	0.001	5.58	44, 6, 42
CN	Medial frontal	86	0.027	4.95	−6, 46, 40
AN	Temporoparietal	531	0	9.71	56, −42, 10
AN	Inferior frontal	194	0.003	9.22	50, 30, 10
AN	Temporal	204	0.003	9.21	−56, −10, −16
AN	Temporal	402	0	8.18	54, −2, −20
AN	Medial frontal	244	0.001	6.6	6, 54, 14
AN	Cingulate	137	0.02	5.89	−2.48, −10
AN	Temporoparietal	111	0.048	5	46, 10, −34
BN	Temporoparietal	2152	0	12.34	50, −58, 2
BN	Temporal	667	0	8.65	54, −10, −14
BN	Fusiform	255	0	7.97	34, −34, −18
BN	Temporal pole	645	0	7.77	−42, 6, −36
BN	Precuneus	638	0	7.5	14, −60, 22
BN	Temporoparietal	1312	0	7.23	−48, −58, 6
BN	Inferior frontal	224	0	6.93	50, 28, 4
BN	Fusiform	282	0	6.12	−36, −50, −18

**Figure 1:**
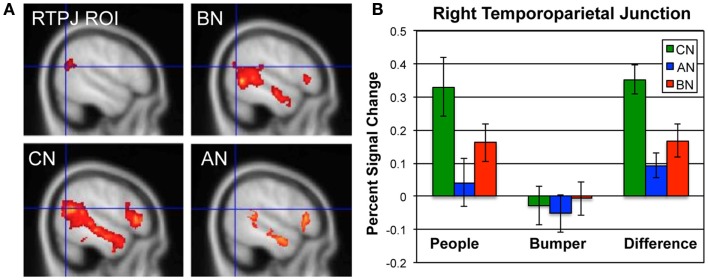
**Neural activations in the RTPJ during the social attribution task**. **(A)** The RTPJ ROI is shown in the upper left, and the group maps for the People–Bumper contrast are all shown at sagittal coordinate *x* = 52 for the BN (upper right panel), CN (lower left panel), and AN (lower right panel) groups. **(B)** The percent signal change in the RTPJ ROI for the People condition, Bumper condition, and the difference in modulation for each group (green, CN; blue, AN; red, BN).

In Figure 1, we show the percent signal change occurring in the ROI for this contrast, the RTPJ, in the CN, AN, and BN groups during the Social Attribution task [means People–Bumper, CN 0.35, AN 0.09, BN 0.17, *F*(50) = 9.7, *p* < 0.001]. The BN group showed significantly less modulation of this region than the CN group [*t*(33) = 2.7, *p* = 0.01; Cohen’s *d* = −0.85; effect size = −0.39] and no difference compared to the AN group [*t*(33) = −1.2, *p* = 0.23]. Similar to the AN group, the differences in activation are primarily the result of less activation of this region during the People condition. We also examined percent signal change by subject group in a MPFC ROI previously described in a similar task as related to outcomes in adolescent AN (37). Although the BN subjects had less activation in this ROIs than the other groups, it was not statistically different from either of the other groups [means, CN 0.30, AN 0.22, BN 0.17, *F*(50) = 2.1, *p* = 0.13].

### Social identity task

The Social Identity Task required subjects to read and respond to social adjectives presented in three different conditions (Self, Friend, and Reflected) in the scanner. For each statement, we obtained a response on a four point scale and a reaction time. There were no significant differences across the three groups in any condition for either average response [mean response, Self: CN 2.53, AN 2.44, BN, 2.49, *F*(51) = 0.178, *p* = 0.84; Friend: CN 2.49, AN 2.46, BN 2.46, *F*(51) = 1.54, *p* = 0.22; Reflected: CN 2.53, AN 2.38, BN 2.41, *F*(51) = 0.75, *p* = 0.48] or the reaction times [mean reaction times in seconds, Self: CN 2.07, AN 2.18, BN 2.26, *F*(51) = 0.35, *p* = 0.70; Friend: CN 2.03, AN 1.97, BN 2.10, *F*(51) = 1.97, *p* = 0.15; Reflected: CN 2.17, AN 2.15, BN 2.16, *F*(51) = 0.42, *p* = 0.66].

**Figure 2:**
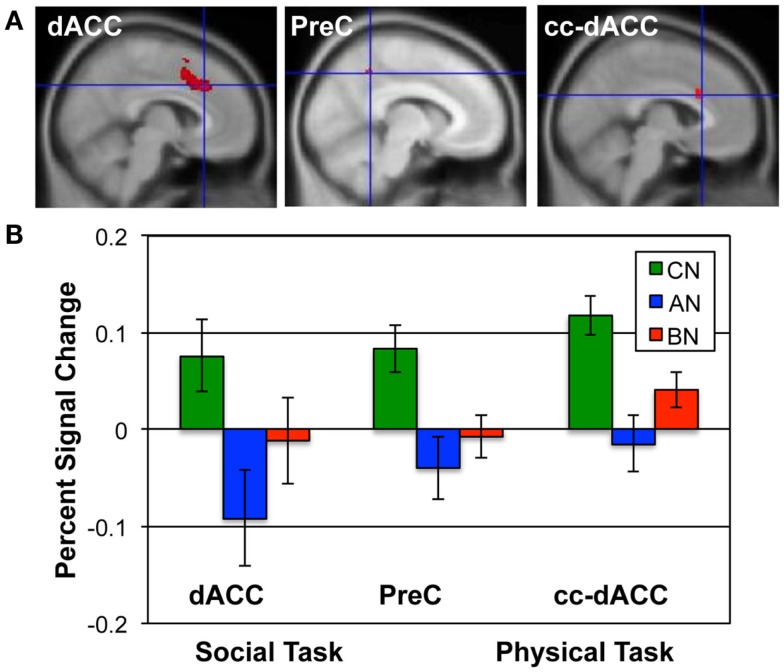
**ROIs and the percent signal change in each group for the Social and Physical Identity contrasts**. **(A)** The upper row shows the extent of each ROI in red; from left to right, the dACC (MNI *x* = 6; Social Identity Reflected–Self); the precuneus (*x* = −8; Social Identity Self–Friend); and the cc-dACC (*x* = −6; Physical Identity Self–Friend). **(B)** The bar graphs shows the average percent signal modulation within each ROI for the CN (green), AN (blue), and BN (red) groups. Percent signal change was computed for the contrast and task that defined each ROI; the Social Identity task for both the dACC and PreC; the Physical Identity task for the cc-dACC.

The Social Identity Task activates regions associated with self-knowledge and personal mentalization. In the personal mentalization contrast (Social Reflected–Self), subjects were asked to imagine what a close friend thinks about their social characteristics in contrast to their own belief about themselves. This contrast differs somewhat from the mentalization processes activated in the Social Attribution task because the mentalization is now attributed to a known individual. The largest clusters of activation occurred in the PreC in all subject groups (Table 3). For both the CN and BN groups, this cluster also included a portion of the posterior Cing, but the AN group had a smaller PreC cluster and an additional cluster in the posterior Cing. The BN and CN groups also had other activation clusters including some consistent with activations seen in the impersonal mentalization task (CN subjects, cluster in left medial temporal gyrus; BN subjects, bilateral clusters in the TPJs). In Figure 2, the BN group showed a lower degree of modulation of the ROI from this task contrast, the dACC, than the CN group [means, Reflected–Self, CN 0.076, BN −0.011, *t*(31) = 2.2, *p* = 0.03, Cohen’s *d* = −0.79, effect size = −0.37], and no difference from the AN group [means, Reflected–Self, AN −0.091, BN −0.01, *t*(31) = −2.0, *p* = 0.06].

**Table 3:** **Clusters in the CN, AN, and BN group maps during the Reflected–Self contrast of the social identity task**.

Group	Region	Volume	Cluster *P*	Peak *T*	MNI *x*, *y*, *z*
CN	Lingual gyrus	151	0.001	8.15	18, −82, −6
CN	Posterior cingulate	1008	0	6.74	8, −24, 28
CN	Middle temporal	135	0.002	6.36	−52, −16, −12
CN	Cuneus	79	0.035	5.83	16, −76, 10
CN	Superior frontal	140	0.002	4.84	−2, 8, 56
AN	Precuneus	137	0.002	6.02	−8, −64, 34
AN	Posterior cingulate	85	0.024	5.44	4, −30, 24
BN	Precuneus	2180	0	12.76	12, −70, 34
BN	Temporoparietal	202	0	8.63	48, −42, 16
BN	Temporoparietal	361	0	6.88	−48, −58, 20
BN	Cingulate	108	0.003	6.31	18, −36, 40

The self-knowledge comparison (Social Self–Friend) led to very different activation patterns in the AN and BN subjects compared to the CN subjects (Table 4). Notably, the CN subjects only activated clusters in the occipital lobes, whereas the AN and BN subjects had many clusters with the largest in occipital, parietal, and frontal cortex. In Figure 2, the BN group also showed significantly less modulation of the ROI from this task contrast, the PreC, than the CN group [means, Self–Friend, CN 0.083, BN −0.001, *t*(31) = 2.7, *p* = 0.01, Cohen’s *d* = −0.94, effect size = −0.43], and no difference from the AN group [means, Social Self–Friend, AN −0.039, BN −0.001, *t*(31) = −0.9, *p* = 0.36]. We also examined percent signal change in the vmPFC and DLPFC but found no differences in either region across the three groups [vmPFC, means CN 0.02, AN 0.02, BN 0.18, *F*(50) = 1.75, *p* = 0.18; DLPFC, means CN 0.08, AN 0.07, BN 0.14, *F*(50) = 1.18, *p* = 0.31].

**Table 4:** **Clusters in the CN, AN, and BN group maps during the Self–Friend contrast of the social identity task**.

Group	Region	Volume	Cluster *P*	Peak *T*	MNI *x*, *y*, *z*
CN	Lingual	915	0	8.7	−4, −94, −4
CN	Lingual	454	0	6.83	8, −82, −4
CN	Occipital	115	0.007	6.13	32, −86, 12
CN	Occipital	290	0	6.11	−24, −68, −18
AN	Occipital	4399	0	8.86	24, −92, −2
AN	Superior parietal	1358	0	8.8	−22, −64, 48
AN	Caudate	95	0.009	7.35	16, 14, −2
AN	Middle frontal	1293	0	7.27	−54, 18, 30
AN	Superior frontal	923	0	7.23	6, 8, 58
AN	Inferior frontal	560	0	6.46	−44, 40, 2
AN	Superior temporal	166	0	6.14	48, 16, −10
AN	Precuneus	539	0	5.97	18, −70, 50
AN	Middle frontal	75	0.03	5.79	36, 32, 28
AN	Inferior parietal	75	0.03	5.71	38, −50, 42
AN	Cerebellum	74	0.032	5.64	10, −76, −44
AN	Inferior frontal	137	0.001	5.04	36, 2, 22
BN	Occipital	839	0	9.3	−12, −92, 14
BN	Parietal	1087	0	8.48	−52, −22, 38
BN	Occipital	979	0	8.13	14, −86, −2
BN	Cingulate	991	0	8.02	4, 8, 64
BN	Precuneus	185	0	7.17	22, −56, 36
BN	Fusiform	216	0	6.46	−36, −62, −18
BN	Precentral	128	0.001	6.46	54, 2, 38
BN	Inferior frontal	482	0	6.33	−56, 12, 22
BN	Precentral	169	0	6.1	48, −16, 42

### Physical identity task

The Physical Identity Task required subjects to read and respond to physical descriptive phrases presented in three different conditions (Self, Friend, and Reflected) in the scanner. For each statement, we obtained a response related to agreeing or disagreeing with the description using a four point scale and a reaction time. There were no significant differences across the three groups in any condition for either average response [mean response, Self: CN 2.39, AN 2.40, BN, 2.36, *F*(51) = 0.178, *p* = 0.84; Friend: CN 2.36, AN 2.40, BN 2.31, *F*(51) = 1.54, *p* = 0.22; Reflected: CN 2.31, AN 2.31, BN 2.24, *F*(51) = 0.75, *p* = 0.48) or the reaction times [mean reaction times in milliseconds, Self: CN 2339, AN 2294, BN 2367, *F*(51) = 0.35, *p* = 0.70; Friend: CN 2392, AN 2267, BN 2440, *F*(51) = 1.97, *p* = 0.15; Reflected: CN 2493, AN 2414, BN 2492, *F*(51) = 0.42, *p* = 0.66].

In the Physical Identity self-knowledge contrast (Physical Self–Friend), very different activation patterns were present in the three groups (Table 5). The CN subjects had several clusters in the anterior and middle Cing; the AN subjects had clusters in the inferior frontal gyri; and the BN subjects showed no activation clusters at all. In Figure 2, the BN group showed no differences in the modulation of the ROI for this task contrast, the cc-dACC, with either the CN group [means Physical Self–Friend, CN 0.12, BN 0.04, *t*(31) = 1.8, *p* = 0.09] or the AN group [means, Physical Self–Friend, AN −0.014, BN 0.04, *t*(31) = −1.3]. We also examined percent signal change in vmPFC and DLPFC but found no differences in either region across the three groups [vmPFC, means CN 0.06, AN 0.04, BN −0.04, *F*(50) = 0.44, *p* = 0.65; DLPFC, means CN 0.06, AN 0.00, BN 0.05, *F*(50) = 1.35, *p* = 0.27].

**Table 5:** **Clusters in the CN, AN, and BN group maps during the Self–Friend contrast of the physical identity task**.

Group	Region	Volume	Cluster *P*	Peak *T*	MNI *x*, *y*, *z*
CN	Anterior cingulate	614	0	7.64	−6, 22, 24
CN	Anterior cingulate	104	0.011	6.23	2, 42, 12
CN	Cingulate gyrus	184	0	5.14	6, −8, 34
AN	Inferior frontal	80	0.022	4.84	40, 16, −16
AN	Medial frontal	70	0.04	4.49	−2, 36, 38
AN	Inferior frontal	70	0.04	4.45	−50, 20, −6
BN	No regions				

## Discussion

Neuroimaging work in the last decade has shown that neural regions involved in self-knowledge are often also activated in social cognitive processing, so the same brain regions that enable understanding one’s own self may also be involved in understanding others (8, 9, 30). One diagnostic criterion for both AN and BN is related to self-knowledge: body shape or weight having undue influence on self-esteem (1). Additionally, problems related to understanding self and others have long been observed in AN (14, 43, 44). Recently, neural evidence of differences in social processing has been reported in AN subjects (36, 37). Here, we assessed whether BN subjects showed more similarities to AN or CN subjects in their neural activations in response to fMRI tasks requiring social processing.

First, it is worth observing that the psychiatric and demographic data for the subjects with AN and BN were only significantly different from each other in that the AN subjects had a lower body mass index. On all other scales, including measures of self-esteem and social behavior, the two subject groups did not differ from one another. There were two differences in comparisons with the CN group on subscales of the SPSI-R: AN subjects had a higher avoidance style and BN subjects showed less rational-problem solving than the CN subjects, but there were no significant differences on these measures in the direct comparisons of the AN and BN subjects. These results are consistent with studies of clinical and personality characteristics in the literature that have examined both AN and BN subjects: few differences are identified, supporting a theory that similar psychological processes underlie both disorders (45–49). Many self-report and clinical measures of psychiatric symptoms depend both upon a subject’s willingness to admit to their symptoms and concerns as well as their ability to recognize and report on their actual symptoms (50). In eating disorders, minimization and denial of symptoms are frequently observed, making psychological and cognitive assessments challenging (51, 52). Neuroimaging data is less likely to be affected by these problems. This study suggests that neural data may provide increased sensitivity for the detection of altered brain function in eating disorders.

Few studies have examined social cognition in BN (19). Interestingly, nearly all of these studies have examined social cognition using facial stimuli, either in a recognition of feelings portrayed by faces (16, 18), an identification of emotions in faces (53, 54), or through an emotional facial Stroop task (48). Amongst these tasks, only the emotional Stroop task, showed strong differences in direct comparisons of BN and CN subjects. Akin to these studies, the neural data from the Social Attribution task showed more similar activation clusters in the BN and CN group maps than in the AN and CN group maps. Notably, both the CN and BN subjects showed significant activations bilaterally in the fusiform face areas in the People–Bumper contrast but the AN group did not have activation in this region. In concert with the numerous behavioral observations of differences related to facial emotion processing in AN (15–17, 55), our neuroimaging data suggest that the neural regions that subserve the processing of facial expressions may be intact in BN but not in the AN (Figure 1; Table 1).

However, the BN subjects did show less modulation than the CN subjects within the RTPJ, the ROI previously identified as showing differences in the task activations using the whole-brain comparisons of the AN and CN groups. This area has been most consistently associated with TOM across a wide variety of imaging tasks that include imagining human movement, interpreting stories, and viewing complex videos (32, 56–59). Our demonstration of reduced modulation of this region in the BN group suggests that there are similarities in the neural processing of TOM in both types of eating disorders. This finding further highlights the fact that neuroimaging markers for cognitive processes may be more sensitive to measuring certain aspects of processing, as the behavioral studies have not detected mentalization differences in BN subjects.

Bydlowski (60) reported reduced TOM in BN subjects using the Levels of Emotional Awareness Scale (LEAS). This is a TOM task that involves answering questions about one’s own emotions and another person’s emotional state based on responses to short vignettes, rather than viewing faces or videos. In PET imaging studies, LEAS scores has been positively correlated with emotional arousal in the dACC in healthy people (61–63) but negatively correlated in post-traumatic stress disorder patients (63). Interestingly, the Social Identity mentalization contrast showed an opposite pattern of modulation in the dACC in the AN and BN groups compared to the CN group. In concert with our data, these results suggest that neural differences in the dACC related to social and emotional processing are present in both AN and BN. Interestingly, activations of the dACC are more commonly observed in tasks with personal relevance (30), a condition present in our Identity task mentalization condition but not the Social Attribution task.

One of the most intriguing findings relates to the differences seen in both eating disorder patients and healthy people with a mentalization process that is personal (*my friend thinks*…) compared to the impersonal task (*People: All friends?*). Very different neural regions are engaged in these two tasks, demonstrating that tasks that separate personal and impersonal mentalization may be important for examination of psychopathology related to social processing. Our neural data implies that the consideration of one’s own self may fundamentally alter social cognitive processing. Recognition of the specific neurocognitive demands of both imaging and behavioral tasks may be essential in detecting psychological and biological differences in eating disorders. Further research with more complicated behavioral and neuroimaging tasks that assess personal and impersonal mentalization are warranted in BN.

Stein and Corte (64) described identity as the stable yet evolving set of memory structures relating to one’s own experiences, and dissociable into different dimensions, which they referred to as self-schemas. They proposed that in eating disorders, the self-schemas related to emotional and physical understanding of one’s own self-state are impaired (64–66). Limitations in assessment of their own emotions are seen in the elevated levels of alexithymia reported in both AN and BN (46, 60, 67–69). Problems in self-esteem are also present and often precede the development of both AN and BN (20–22, 70, 71). The presence of negative self-beliefs unrelated to shape and weight has been proposed as a core component of eating disorders (26, 27).

In Social Identity self-knowledge contrast (Social Self–Friend), the group maps of the AN and BN subjects were very different from the CN subjects. The CN subjects only activated areas in the occipital and lingual cortex, whereas the AN and BN subjects showed significant activations not only in those areas, but also in frontal, parietal, and temporal regions (Table 4). Additionally, we observed reduced activation of the dorsal PreC in AN and BN subjects, the area previously identified with greater modulation in the CN subjects than the AN. Consistent with our findings, reduced modulation of the PreC has been reported in two other imaging tasks in BN. Ashworth and colleagues (72) examined cognitive processing related to social emotional appraisals by asking subjects to remember and match negative facial expressions, and Pringle and colleagues (28) asked subjects to consider whether negative eating and depression words were self-relevant or not. Together with our data, these studies support an idea that PreC activity in response to self-evaluation may be altered in eating disorders. The PreC has connections with temporal, limbic, and parietal regions, suggesting that it serves to integrate current physical and emotional status with prior experiences (73). The reduced modulation of the PreC in the eating disorder subjects observed here implies a reduced connection between physiological state and personal experience, supporting an idea that the psychological processes that mediate identity formation are disrupted in eating disorders.

In the physical self-knowledge contrast, subjects were asked to think about their own physical appearance. This task strongly activated a ventral region of the dACC, immediately adjacent to the corpus callosum in the CN subjects, with little modulation in the AN subjects, and no clusters identified in the BN subjects. In this comparison, BN subjects were not significantly different from either the AN or the CN subjects. The variance of the BN group was nearly twice that of either the AN or CN group for this ROI, supporting an idea that some subjects with BN may have problems activating this cortical area and others may not. For the other ROIs, the variance of the BN group was similar to that of the AN and CN groups. Interestingly, Marsh and colleagues have focused on the neural circuits involved in self-regulation in BN, and also reported differences in the activation of this area of the dACC in BN (74, 75). From a cognitive perspective, the differences in the cc-dACC suggests that some subjects with BN, but not all, think about their current physical or physiological state differently than healthy people. This neural difference may correspond to less information about physiological needs being available in the minds of eating disorder patients, making it easier for these individuals to develop feeding behaviors that are removed from nutritional needs. Future studies may want to focus on whether the activation of the cc-dACC can be related to psychological measures of body shape perception and interoceptive awareness.

Recently, Schulte-Ruther and colleagues (37) used an fMRI task similar to the Social Attribution task to examine whether longitudinal changes in social cognitive regions were associated with weight recovery in adolescents being treated for AN. They observed reduced activation of temporal and medial frontal regions both before and after weight recovery in AN subjects compared to CN subjects. They also reported that stronger modulation of one social cognition region, the MPFC at the start of treatment, was predictive of outcome. We also examined responses in this MPFC ROI, but found no differences in the three subject groups. One major difference in the studies is that all our subjects were at a stable weight when scanned, whereas the earlier study had observed changes in this region related to outcomes following treatment. Nevertheless, the observation that MPFC may be relevant to recovery is particularly exciting when considered in the context of a study by Somerville and colleagues (76) in which healthy people with low self-esteem showed modulation of responses in their vACC and MPFC in response to social feedback whereas people with high self-esteem showed no changes in this region in response to feedback. Low self-esteem has previously been shown to be predictive of the development of eating disorders (20, 22, 25) as well as an indicator of outcome and severity (77, 78). Neural responses within the ACC and MPFC to social feedback may provide a biological mechanism that connects social cognitive responses and self-esteem with eating pathology; understanding how biological factors impact specific patients may lead to improved outcomes by providing more individualized treatments in the future.

Additionally, two earlier studies have identified frontal cortical regions associated with eating disorders and processing verbal stimuli. Pringle and colleagues (28) found differences in dorsolateral PFC in response to negative emotional words considered in the context of oneself, and Miyake et al. (42) has reported differences in vmPFC associated with responses to selecting a negative body image words compared to selecting the most neutral of a random word sets. Although we did not observe differences across our subject groups in these same regions in either the Social Identity task or the Physical Identity task, this is likely related to differences in task design. Our subjects performed Self-appraisals, Friend-appraisals, and Reflected-appraisals, using the same sets of adjectives. As such, neither of the studies showing effects in frontal regions had a comparison situation involving the same stimuli words referenced to a different person. This suggests that the areas we have identified in the dACC, cc-dACC, and PreC may be specific for altered cognitions related to one’s own self in eating disorder subjects, whereas frontal activation differences may relate to the cognitions evoked by physical and emotional descriptive terms.

There are a number of limitations to these studies. First, the sample groups were small and as such the study may be underpowered to identify both differences and similarities that are present. Furthermore, the BN group showed more variability in their neural activations than the AN and CN groups, and that variability may warrant collecting a larger group to identify specific differences. Additionally, a larger study could explore the relationships between neural activity and clinical symptoms for both AN and BN. Potentially, neural data may provide an additional tool to assess the severity and symptoms present in a specific patient with an ED.

Another limitation of any single-time point psychiatric study is that the presence of neural differences does not determine whether these differences are a cause or an effect of the disorder. In eating disorders, medical issues are likely to alter brain function. Purging behaviors alter electrolytes, a critical factor for neuronal signaling (79). Restriction leads to nutritional deficiencies and hormonal changes, additional factors that alter brain function (80). Neurodevelopment is critically dependent on myelination (81, 82), and that process may be impacted with the presence of an eating disorder in adolescence and young adulthood. The size of the anterior Cing, a brain region fundamental in self-processing, decreases with the severity of starvation in AN (83). Our studies show differences in neural activations in response to social tasks in patients currently or recently with eating disorders, including both AN and BN.

Differences in neural activations in psychiatric populations may be a result of a variety of processes. They can emerge because of pre-existing biological differences that lead to the disorder, because of the effects of having the disorder such as electrolyte changes, or may merely be a reflection of current psychological differences related to processing stimuli relevant to the disorder. Most commonly, differences are viewed as a biological predisposition to the illness but it is impossible to determine if these differences existed before the eating disorder and if they will still be present following recovery from the eating disorder. Our data show that there are differences in the neural activity that underlies social processing in people with BN. Clinically, this implies that social processing pathways, including TOM and self-knowledge, are engaged differently during an eating disorder. This reinforces choosing treatment models with a focus on issues related to social interaction and function in addition to disordered eating behaviors (84–86). Neural evidence of social processing differences in eating disorders may be important in helping patients accept treatments that appear indirectly related to alteration of eating patterns.

In summary, these experiments examined the neural modulations in response to fMRI tasks focusing on self-knowledge and social cognition in BN. We observed modulation in the BN group that was consistently intermediate between the AN and CN groups. In three ROIs, all of which were activated in CN during MRI tasks involving social evaluation, the BN subjects were significantly different from the CN subjects but not from the AN subjects. This suggests that neural processes that mediate social thinking are similar in AN and BN. Recently, Lavender and colleagues (87) examined outcomes for BN using a group therapy focusing on emotional and social mind training, and found recovery rates similar to more established cognitive behavior therapy. That study, in concert with our neural data, suggest that further exploration of social processing interventions may lead to improved outcomes in BN. One interesting observation in that pilot treatment study was that subjects in the emotional and social training group were more likely to attend sessions, suggesting that this type of treatment may help to engage patients in treatment. The fourth region, cc-dACC, was identified in a contrast of the Physical Identity task. In this area, the BN subjects were not significantly different from either the AN or the CN subjects. This suggests that cognitions surrounding physical appearance may be altered less in BN than in AN, or less consistently altered amongst patients with BN. Overall, our studies demonstrate similarities in neural processing in BN and AN, and suggest that there may be shared biological mechanisms related to processing social concepts that differ systematically from neural modulations seen in healthy CN subjects.

## Conflict of Interest Statement

The authors declare that the research was conducted in the absence of any commercial or financial relationships that could be construed as a potential conflict of interest.

## Footnotes

^1^www.fil.ion.ucl.ac.uk/spm

^2^http://www.mathworks.com

^3^http://www.alivelearn.net/xjview8/

^4^sourceforge.net/projects/marsbar
